# Validation of an HPLC–MS/MS Method for the Determination of Plasma Ticagrelor and Its Active Metabolite Useful for Research and Clinical Practice

**DOI:** 10.3390/molecules26020278

**Published:** 2021-01-08

**Authors:** Jennifer Lagoutte-Renosi, Bernard Royer, Vahideh Rabani, Siamak Davani

**Affiliations:** 1 Service de Pharmacologie Clinique et Toxicologie, Centre Hospitalier Universitaire de Besançon, F-25000 Besançon, France; jennifer.lagoutte_renosi@univ-fcomte.fr (J.L.-R.); broyer@chu-besancon.fr (B.R.); 2 EA 3920, Université Bourgogne Franche-Comté, F-25000 Besançon, France; v.rabani@gmail.com

**Keywords:** ticagrelor, antiplatelet agents, high performance liquid chromatography, tandem mass spectrometry, adverse events, therapeutic drug monitoring

## Abstract

Ticagrelor is an antiplatelet agent which is extensively metabolized in an active metabolite: AR-C124910XX. Ticagrelor antagonizes P2Y12 receptors, but recently, this effect on the central nervous system has been linked to the development of dyspnea. Ticagrelor-related dyspnea has been linked to persistently high plasma concentrations of ticagrelor. Therefore, there is a need to develop a simple, rapid, and sensitive method for simultaneous determination of ticagrelor and its active metabolite in human plasma to further investigate the link between concentrations of ticagrelor, its active metabolite, and side effects in routine practice. We present here a new method of quantifying both molecules, suitable for routine practice, validated according to the latest Food and Drug Administration (FDA) guidelines, with a good accuracy and precision (<15% respectively), except for the lower limit of quantification (<20%). We further describe its successful application to plasma samples for a population pharmacokinetics study. The simplicity and rapidity, the wide range of the calibration curve (2–5000 µg/L for ticagrelor and its metabolite), and high throughput make a broad spectrum of applications possible for our method, which can easily be implemented for research, or in daily routine practice such as therapeutic drug monitoring to prevent overdosage and occurrence of adverse events in patients.

## 1. Introduction

Ticagrelor is an antiplatelet agent belonging to the cyclopentyltriazolo-pyrimidine class [1]. It is extensively metabolized by CYP3A4 and 3A5 in an active metabolite: AR-C124910XX [2]. Ticagrelor reversibly and non-competitively antagonizes P2Y12 receptors at a different site than adenosine diphosphate (ADP) [3], contributing to the antiplatelet effect. The receptor thus remains inactivated and ADP signaling is inhibited. Recently, additional mechanisms of action of ticagrelor have been uncovered. It inhibits adenosine uptake by red blood cells via equilibrative nucleoside transporter 1 inhibition [4]. Moreover, in vitro, ticagrelor limits the transport activity of the cardiac sodium/exchanger NCX1 [5]. Additional effects of ticagrelor, such as an increase in plasma adenosine levels, were initially linked to specific side effects such as bradycardia and dyspnea [6,7]. However, a recent prospective study [6] confirmed that plasma levels of ticagrelor were 2- to 3-fold higher in patients with ticagrelor-related dyspnea compared to those without dyspnea, both after loading and maintenance doses. Interestingly, this effect was observed without any change in adenosine levels. Side effects of ticagrelor were dose-dependent, with a reduction in symptoms observed after discontinuation of ticagrelor [7]. These data pave the way for therapeutic drug monitoring. However, before establishing such a practice, it is important to investigate the relationship between plasma levels of ticagrelor and its active metabolite, and the physiological effects in real-life treated patients. To this end, we developed a reliable and rapid method validated according to the latest Food and Drug Administration (FDA) guidelines to simultaneously determine both ticagrelor and its active metabolite. The method is suitable for pharmacokinetics/pharmacodynamics studies and clinical practice, with a wide-range calibration curve to enable the determination of high concentrations. The method was successfully applied to patient blood samples for a population pharmacokinetics/pharmacodynamics study (NCT03658005) and could easily be implemented in daily routine practice for patient management to prevent overdosage.

## 2. Results

Early during the development of this method, we tested two ionization modes (positive and negative mode) on our heat electrospray ionization (H-ESI) ion source, and the positive mode yielded a better response than the negative mode. Moreover, [^2^H_7_]-ticagrelor, and [^2^H_7_]-AR-C124910XX were judged as the most appropriate internal standards (IS) due to their chemical structure being identical to that of the therapeutic molecules. Quantification was performed using multiple reaction monitoring and different *m*/*z* transitions for quantification and ion ratio confirmation purposes.

### 2.1. Chromatogram and Retention Times

**Figure 1:**
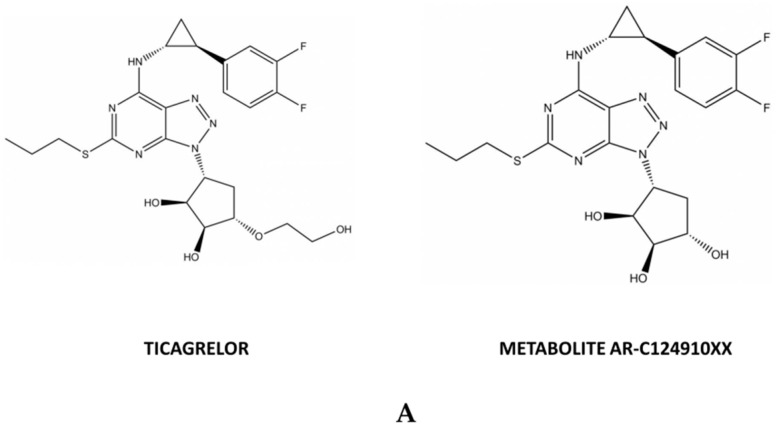

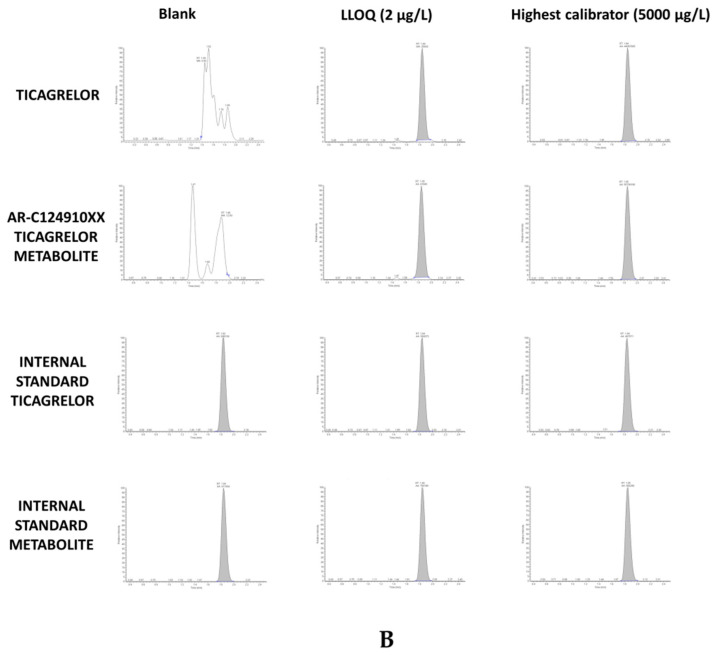
(**A**) Chemical structures of ticagrelor and its active metabolite AR-C124910XX. (**B**) Typical chromatograms of the analytes in blank human plasma samples, at 2 µg/L, the lower limit of quantification (LLOQ), at 5000 µg/L the highest concentration from calibration curves. For each analyte, the chromatograms were obtained from the highest intensity used for quantification.

With our chromatographic conditions, retention times (RT) were 1.8 min for ticagrelor, [^2^H_7_]-ticagrelor, metabolite AR-C124910XX, and [^2^H_7_]-AR-C124910XX (Figure 1B). The total run time was 2.5 min. The RT was similar for all molecules due to the close chemical structure between ticagrelor and its active metabolite (Figure 1A). However, mass spectrometry with selection of the *m*/*z* parent ion allows good identification without interference of each molecule.

### 2.2. Selectivity

Analysis of blank samples showed no interfering peaks at the RT of ticagrelor, metabolite AR-C124910XX, and their respective IS in human plasma. Moreover, signal responses observed for these blank samples could not be visually distinguished from background noise.

### 2.3. Lower Limit of Quantification, Calibration Curve, and Linearity

Our method was linear from 2 to 5000 µg/L for ticagrelor and its metabolite. Considering the calibration curves, deviations of the calculated concentration from the nominal concentration considering each standard sample were below FDA recommendations, 20% for the lower limit of quantification (LLOQ) and 15% for other standard samples. For the pharmacokinetics/pharmacodynamics study purposes, the LLOQ was set at 2 µg/L for ticagrelor and its active metabolite (precision of 13.4% and 19.9%, respectively). Precision at this concentration was below the FDA recommendations of 20%.

### 2.4. Accuracy and Precision

Precision and accuracy obtained during our intra-assay and inter-assay experiments are summarized in Table 1. Six samples were analyzed per day, on three separate days by three different operators. Precision and accuracy were in accordance with the FDA recommendations.

### 2.5. Recovery and Matrix Effect

The extraction recoveries at LLOQ, QC1 (Quality Control 1), QC2, and QC3 were 63.5%, 59.3%, 62.3%, 61.5%; and 50.4%, 57.1%, 62.7%, 67.0% for ticagrelor and metabolite AR-C124910XX, respectively. Relative standard deviation (RSD) of internal standard normalized ratios of ticagrelor and its metabolite were 11.2% and 8.4%, respectively, for low concentration and 13.9% and 4.4%, respectively, for high concentration. No significant matrix effect was present for either molecule.

### 2.6. Stability

In whole blood, both molecules remained stable for 3 h but were not stable for 6 and 24 h at room temperature or at 4 °C (Table 2). After 3 freeze–thaw cycles at −20 °C, the levels of ticagrelor and its metabolite remained stable. Short-term stability in human plasma at room temperature was adequate for ticagrelor but not for its metabolite, in particular for lower concentrations with a RE greater than 15 but less than 20%. After processing, extracts were stable at 4 °C for 72 h.

### 2.7. Contamination

Contamination observed using our method was very low, 0.0007% for ticagrelor and 0.0006% for metabolite AR-C124910XX.

### 2.8. Application to Pharmacokinetics Study

**Figure 2:**
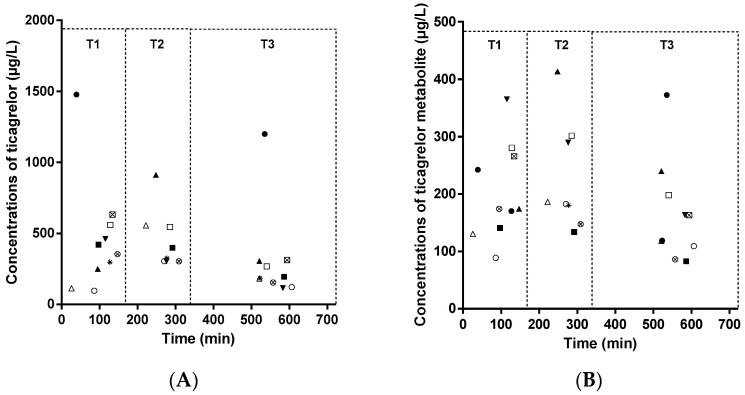
Plasma concentrations versus time: (**A**) ticagrelor (**B**) metabolite AR-C124910XX for *n* = 28 blood sampling in 10 patients after multiple oral administrations of 90 mg bid ticagrelor; with T1: between 0 and 180 min, T2: between 180 and 360 min, T3: between 360 and 720 min. The same symbol is used for the same patient in both charts.

Our validated LC–MS/MS (liquid chromatography–mass tandem spectrometry) method was successfully applied to 28 patient samples (Figure 2). Samples were taken from patients during the maintenance dose of 90 mg ticagrelor twice a day orally. Mean concentrations of ticagrelor were 466 ± 396; 456 ± 212; 304 ± 322 µg/L for measurement between 0 and 180 min (T1 with median at 106 min), 180 and 360 min (T2 with median at 277 min), and 360 and 720 min (T3 with median at 548 min), respectively, whereas mean concentrations of metabolite were 203 ± 83; 229 ± 96; 165 ± 88 µg/L for measurement at the same timepoints as for ticagrelor, during the pharmacokinetics study. Among the results, the highest concentrations (>1000 µg/L) of ticagrelor at T1 and T3 were related to the same patient.

## 3. Discussion

In this paper, we describe a new methodology to simultaneously determine ticagrelor and its active metabolite AR-C124910XX in human plasma by isocratic LC–MS/MS. This method presents several major advantages over other methods previously described in literature: (i) rapidity, (ii) simplicity, and (iii) the wide range of the calibration curve. Furthermore, we show that ticagrelor and its metabolite were not stable for more than 3 h in whole blood at room temperature, or at 4 °C, requiring a rapid isolation of plasma.

Only three methods were completely validated according to 2013 FDA guidelines [8,9,10]. Our method was validated with the last update of the FDA guidelines published in 2018, which include assessment at 4 QC levels (LLOQ, low, middle, high), for accuracy and precision [11]. All acceptance criteria, namely accuracy, precision, matrix effect, and contamination were validated. Extraction recoveries were tested according to the FDA guidelines but were not particularly relevant in our case because we used protein precipitation rather than classical liquid–liquid extraction, with an internal standard, which corrects possible deviation during precipitation or injection into the chromatographic system. The matrix effect was additionally assessed according to European Medicines Agency (EMA) guidelines to complete our assessment of possible interference, with no significant interference observed. To approach conditions of daily practice, the stability of ticagrelor and its metabolite in whole blood were assessed, after 3, 6, and 24 h at room temperature or 3, 6, and 24 h at 4 °C. Assessment of stability in whole blood is important for correct pre-treatment of the sample, to prevent deterioration of the drug, but actually rarely detailed in literature. Both molecules remained stable in whole blood for 3 h at room temperature, but without rapid isolation of human plasma, ticagrelor was not stable in whole blood for 6 and 24 h, even when stored at 4 °C. Thus, concerning stability of the compounds in whole blood, it is not the temperature of storage that induced a bias in response of the molecules, but rather the storage time. Sillèn et al. also showed sufficient stability for transport and storage at room temperature for 1 and 4 h before processing [12]. In contrast, stability in refrigerated storage at 4 °C for 24 h had never been previously assessed prior to our study, to the best of our knowledge. However, ticagrelor and its metabolite were not stable enough to tolerate storage for 6 and 24 h at 4 °C in whole blood. Isolation of plasma and then freezing is essential to avoid deterioration of ticagrelor. This is important to consider for use in research or in clinical practice, to avoid underestimating the levels of ticagrelor and its metabolite.

Our method presents several advantages over other methods previously described for human plasma [8,9,10,13,14,15,16,17], especially for application in daily routine practice. First, our method has a relatively faster run time (2.5 min) compared to other methods. Xu et al. [9], Zhong et al. [18], and Danielak et al. [16] developed methods with 3.4, 4.5, and 8 min of run time, respectively. Only Chae et al. [10] and Sillén et al. [12] obtained a faster run than our technique with 1.6 and 2 min. Second, our analytical method using simple protein precipitation for sample preparation and the use of an isocratic mobile phase is a further advantage for rapid application. Taken together, these properties enable extraction, analysis, and delivery of results for patients within a single day. Indeed, several other methods have used more complex sample preparation, with liquid–liquid extraction [10,18,19], or complex gradient of mobile phase [9,14,15], increasing the overall analysis time. Third, for application in pharmacokinetics studies or in routine practice, the small volume of plasma required for analysis (150 µL), and the wide range of the calibration curve (2–5000 µg/L) are also advantages compared to previously described methods [8,9,10,13,16,17,18]. Indeed, in several methods, for ticagrelor, the range was limited up to 800 [9], 1500 [10], and 2000 µg/L [13,18]. Sillén et al. [12] reported a range up to 5000 µg/L for ticagrelor, but the range remained lower for its metabolite, at 2500 µg/L. Hobl et al. [14] presented the same range as our technique for both molecules. The highest calibrator level in our method makes it possible to investigate the link between high concentrations of ticagrelor or its metabolite, and possible adverse events. Several authors have described methods with a lower limit of quantification. Three methods obtained an LLOQ at 0.5 [18], 0.781 [9], and 1.25 µg/L [16] and performed better than our method, at 2 µg/L. However, the upper limit of quantification was lower, i.e., 2000 µg/L for the maximum upper limit reached with these methods [9,17,19]. In routine practice, achievement of a better lower limit of quantification is not very useful and often involves more complex and time-consuming extractions.

Our method was developed for a population pharmacokinetics/pharmacodynamics study, but also for the purpose of daily routine practice, to improve our understanding of the side effects associated with ticagrelor. In this regard, plasma concentration–time profiles of ticagrelor and metabolite AR-C124910XX for 28 patient samples after multiple oral administrations of 90 mg bid ticagrelor were successfully determined. Interestingly, mean concentration of ticagrelor at T1 (between 0 and 180 min) in patients without adverse events was 466 ± 396 µg/L, which is similar to the concentrations described in a recent study [6] for patients without ticagrelor-related dyspnea. Indeed, Ortega et al. found a ticagrelor concentration of 493 µg/L [267–629 µg/L], 1 to 2 h after a maintenance dose. No significant adverse events were reported in patients included in our pharmacokinetics study. Therefore, our data confirm this range of concentrations during the absorption phase of ticagrelor in patients without ticagrelor-related dyspnea. Higher concentrations have been found in case of adverse events, in a range of 844–2330 µg/L after a maintenance dose, with a maximal concentration of 3670 µg/L after ticagrelor loading in a patient who presented dyspnea [6], hence the relevance of a high calibrator at 5000 µg/L for our method. In this context, when blood sampling is performed just after loading, higher concentrations can be determined, without dilution of the sample preparation. Drug–drug interactions between ticagrelor and its active metabolite during concomitant treatment, such as atorvastatin [16], morphine [14], or cyclosporin [19], have also been described. Thus, the method presented here is especially suitable for high concentrations observed during adverse events or drug–drug interactions, contrary to other, previously described methods. The LC–MS/MS method described here could be applicable in a range of common situations, such as pharmacokinetics studies or documentation of side events with ticagrelor.

Finally, therapeutic drug monitoring needs study in a clinical setting to potentially assess a link between drug concentrations and its clinical effects. Such study implies analytical methods to assess drug concentrations in particular clinical situations, e.g., in the present case when drug concentration is high. For such purposes, our method is of particular interest as we can rapidly and simply measure ticagrelor and its metabolite concentrations with a wide range of concentrations, including those potentially leading to adverse effects. If such a link between ticagrelor and metabolite concentrations and adverse effects is observed, the same method will be useful to perform their therapeutic drug monitoring.

## 4. Materials and Methods

### 4.1. Chemical and Reagents

Powder of ticagrelor, and its active metabolite AR-C124910XX (Figure 1) and their respective stable isotope-labeled Internal Standards (IS) [^2^H_7_]-ticagrelor, and [^2^H_7_]-AR-C124910XX were purchased from Alsachim^®^ (Illkirch, France). Mass spectrometry (MS)-grade water and acetonitile were purchased from Carlo Erba^®^ (Val de Reuil, France). Analytical-grade formic acid was purchased from Sigma Aldrich^®^ (St. Quentin Fallavier, France). Non-therapeutic blank human plasma was obtained from the French blood transfusion center (EFS Bourgogne Franche Comté, Besançon, France).

### 4.2. Standard and Quality Control Sample Preparation

Individual stock solutions of ticagrelor, AR-C124910XX and their respective IS were prepared at a concentration of 500 mg/L in MS-grade acetonitrile. Stock solutions were stored at −80 °C. Intermediate and final working solutions were extemporaneously prepared using serial dilutions in acetonitrile. Standard samples and quality control (QC) samples were prepared in blank human plasma by adding the appropriate amount of ticagrelor or active metabolite AR-C124910XX. Calibrators were of 2, 10, 50, 100, 350, 1000, and 5000 µg/L and QC levels were of 8, 800, and 3500 ng/L, for, respectively, QC1, QC2, and QC3.

### 4.3. Sample Extraction

First, 150 µL of plasma (patients, calibrators, or QC samples) was mixed with 320 μL of acetonitrile containing the IS at a concentration of 600 µg/L. The mixture was vortex-mixed for 30 s, then centrifuged at 10,000 rpm. Then, 350 μL of supernatant was transferred into tubes for evaporation to dryness under nitrogen flow. The residue was reconstituted in 150 µL of acetonitrile/water (85:15; *v*/*v*; 0.1% formic acid) and 5 µL was injected into the chromatographic system.

### 4.4. Instrument and Analytical Conditions

The Accela^®^ chromatographic system including Accela^®^ Pump and Autosampler was used (Thermo Scientific^®^, Waltham, MA, USA). Chromatography was achieved with a Zorbrax SB-C8 3.0 × 150 mm; particle size 3.5 µm (Agilent^®^, Les Ulis, France) maintained at a temperature of 30 °C and with a constant flow rate of 450 μL/min. An isocratic mobile phase was used with a mixture of acetonitrile and water at 0.1% formic acid (85:15; *v*/*v*). The mass spectrometry analyses were performed using a Finnigan TSQ Quantum Ultra^®^ MS/MS mass spectrometer (Thermo Scientific^®^, Waltham, MA, USA) operating with a heat electrospray ionization (H-ESI) ion source set on positive mode. Optimized mass spectrometer parameters were as follows: spray voltage at 3500 V; vaporizer temperature: 200 °C; sheath gas pressure: 30 psi; ion sweep gas pressure: 2 arbitrary units (au); auxiliary gas pressure: 15 au; capillary temperature: 400 °C; collision pressure: 1.5 mTorr. The determination of *m*/*z* transitions and operating conditions for each compound are presented in Table 3.

### 4.5. Analytical Data Processing

LCQuan^®^ 2.5.6 and Xcalibur^®^ 2.0.7 (Thermo Scientific^®^, Waltham, MA, USA) software was used to process chromatographic and MS data. GraphPad Prism^®^ 6.01 was used for data analysis.

### 4.6. Validation Procedure

The method was validated according to the FDA guidelines of May 2018 [8]. The following aspects were investigated in the validation process: selectivity, calibration curve, lower limit of quantification, intra-assay and inter-assay accuracy and precision, recovery, matrix effect, sample stability in different conditions, and contamination.

#### 4.6.1. Selectivity

Ten different blank human plasma samples were used to investigate interferences. After extraction and analysis, chromatograms were visually checked to assess interferences.

#### 4.6.2. Calibration Curves

For each experiment, calibration curves for ticagrelor and metabolite AR-C124910XX were performed on blank human plasma. They were obtained using linear least-squares regression with 1/X^2^ weighting between calibrator concentrations and measured area peak ratio ticagrelor/IS or metabolite/IS. The calculated concentrations of the calibrators should be within ±15% of their nominal values, except for the LLOQ for which they should be within ±20%. At least six calibrators must fulfil this criterion to accept the calibration curve.

#### 4.6.3. Determination of the Lower Limit of Quantification

The LLOQ was checked by assessing intra-assay (*n* = 10) accuracy with a relative standard deviation (RSD) less than 20% for 2 µg/L of ticagrelor and metabolite AR-C124910XX.

#### 4.6.4. Accuracy and Precision

Intra- and inter-assay accuracy and precision were determined on LLOQ, QC1, QC2, and QC3 samples (*n* = 6/per day), on three separate days by three different operators. Precision and accuracy are expressed with RSD and relative error (RE), respectively. For acceptable precision and accuracy, variation of RSD or nominal concentration should be below 15% respectively, except for the LLOQ, where the variation was permitted to be up to 20%.

#### 4.6.5. Recovery and Matrix Effect

The extraction recovery of ticagrelor and the metabolite AR-C124910XX were performed in six replicates by comparing samples at LLOQ, QC1, QC2, and QC3 versus extracts of blanks spiked with ticagrelor and metabolite AR-C124910XX post extraction. The matrix effect was also assessed, according the EMA guidelines, because FDA guidelines do not make formal recommendations on this point. The matrix effect was investigated using six lots of blank matrices from individual donors. The matrix factor was calculated as the ratio of the peak area of ticagrelor or its metabolite in plasma of blank plasma from six individual donors spiked after extraction with ticagrelor and its metabolite, to the peak area of both molecules in a pure solution of acetonitrile. The internal standard normalized matrix factor was calculated by dividing the matrix factor of ticagrelor or the metabolite by the matrix factor of their respective internal standard. The RSD of this internal standard normalized matrix factor should be under 15%, for a low and high level of concentration.

#### 4.6.6. Stability

The stability of ticagrelor and its active metabolite AR-C124910XX in human plasma or whole blood under different common situations encountered were assessed via analysis of low and high levels of QC, patient samples, or spiked whole blood for 3 freeze–thaw cycles at −20 °C; bench-top at room temperature; extracts at 4 °C for 72 h after processing; whole blood at room temperature for 3, 6 and 24 h; whole blood at 4 °C after 3, 6 and 24 h. Both molecules were considered to be stable in plasma samples when RE was below 15% of the nominal concentrations.

#### 4.6.7. Contamination

As the FDA guidelines do not recommend a specific method for assessing contamination, our inter-sample contamination (due to the autosampler carry-over) was tested following the recommendations of the COFRAC (French Accreditation Committee) [9]. We analyzed sequences of samples: three high-concentration samples of quality control (QC) (QC3a, QC3b, QC3c) then three low-concentration samples (QC1a, QC1b, QC1c). This process was repeated four times and calculated using the following formula, where “m” stands for mean: mQC1a, mQC1c, mQC3, mQC1c were mean of QC1a, mean of QC1c, mean of all QC3, and mean of QC1c, respectively.

$$\mathrm{Contamination}\;\left(\%\right)=\;\frac{\left(\mathrm{mQC}1a\;-\;\mathrm{mQC}1c\right)}{\left(\mathrm{mQC}3-\mathrm{mQC}1c\right)}*100$$

The absence of carry-over was defined as a contamination <1%.

#### 4.6.8. Application to Pharmacokinetics Study

Ten patients who had previously given informed consent and who had been treated with ticagrelor were included for a population pharmacokinetics/pharmacodynamics study (NCT03658005). Blood samples were obtained then rapidly centrifuged and plasma samples were frozen at −20 °C for later measurement. Plasma drug concentrations were determined using our analytical method.

## 5. Conclusions

In summary, this HPLC–MS/MS method for the simultaneous determination of ticagrelor and its active metabolite AR-C124910XX in human plasma is simple, rapid, and validated according to the latest FDA guidelines update. The wide range of the calibration curve (i.e., 2–5000 µg/L) for both molecules enables a broad spectrum of applications, such as bioequivalence, clinical studies, or routine practice, especially for the investigation of adverse events or drug–drug interactions. In the future, therapeutic drug monitoring of ticagrelor could be generalized to prevent overdosage and the occurrence of adverse events.

## Figures and Tables

**Table 1:** Intra- and inter-assay precision and accuracy for ticagrelor and its metabolite AR-C124910XX.

				Intra-Assay		Inter-Assay	
Molecules		Spiked Concentration (µg/L)	Nominal Concentration (µg/L)	Precision (%, RSD)	Accuracy (%, RE)	Precision (%, RSD)	Accuracy (%, RE)
Ticagrelor	LLOQ	1.8	2	13.4	−8	19.9	−11.5
	QC1	7.9	8	5.4	−4.5	9.9	−1.6
	QC2	768.5	800	3	−2.8	4.9	−3.9
	QC3	3313.2	3500	4.5	−4	4.6	−5.3
Active Metabolite AR-C124910XX	LLOQ	1.8	2	14	4.5	19.4	−12
	QC1	8.3	8	2.3	8.4	5.9	3.6
	QC2	780	800	4.2	−3.7	4	−2.5
	QC3	3396.7	3500	6.7	−0.9	6.1	−3

Results are expressed as the mean of measured concentrations (*n* = 6 per day/3 days) with RSD: relative standard deviation (%), RE: relative error (%), LLOQ: lower limit of quantification, QC: quality control.

**Table 2:** Stability of ticagrelor and its metabolite AR-C124910XX.

Molecules	Storage Condition	Type of Samples	RSD (%)	RE (%)
Ticagrelor	Whole blood at room temperature for 3 h	Low level	0.4	−3
		High level	7	0.2
	Whole blood at room temperature for 6 h	Low level	48.3	−1.5
		High level	1.8	22
	Whole blood at room temperature for 24 h	Low level	3.9	−16.1
		High level	14.4	−20.6
	Whole blood at 4 °C for 3 h	Low level	4.9	5.4
		High level	5.7	10.9
	Whole blood at 4 °C for 6 h	Low level	4.5	−14.2
		High level	27.6	−14.9
	Whole blood at 4 °C for 24 h	Low level	4.5	23.3
		High level	0.3	27.4
	Three freeze-thaw cycles at −20 °C	QC1	4.6	4
		QC3	2.6	−4.2
	Plasma on bench-top at room temperature for 24 h	QC1	5.2	6.2
		QC3	1.4	−3.2
	Processed samples at 4 °C for 72 h after extraction	28 samples	NA	3.7
Active Metabolite AR-C124910XX	Whole blood at room temperature for 3 h	Low level	1.4	1.8
		High level	3.7	−0.3
	Whole blood at room temperature for 6 h	Low level	60.2	4.6
		High level	12.2	29.8
	Whole blood at room temperature for 24 h	Low level	2	−18.9
		High level	9.6	−19.6
	Whole blood at 4 °C for 3 h	Low level	3.9	5
		High level	0.9	−0.9
	Whole blood at 4 °C for 6 h	Low level	2.4	−23.3
		High level	12.5	−13.1
	Whole blood at 4 °C for 24 h	Low level	11.7	1.4
		High level	0.1	7
	Three freeze-thaw cycles at −20 °C	QC1	5.1	−14
		QC3	3	0.03
	Plasma on bench-top at room temperature for 24 h	QC1	7.7	−19.2
		QC3	8.4	−1.9
	Processed samples at 4 °C for 72 h after extraction	28 samples	NA	13.8

RSD: relative standard deviation (%), RE: relative error (%), QC: quality control.

**Table 3:** Mass spectrometry operating conditions for our compounds. Transitions (*m*/*z*) used for quantification are labeled *.

Compound	Parent Ion (*m*/*z*)	Product Ion (*m*/*z*)	Collision Energy (eV)	Tube Lens Voltage (V)
Ticagrelor	523.225	152.960 *	35	95.36
		132.94	35	95.36
AR-C124910XX	479.192	132.970 *	44	151.66
		479.192	59	151.66
[^2^H_7_]-ticagrelor	530.283	133.1	62	115.38
[^2^H_7_]-AR-C124910XX	486.238	133.01	47	86.85

## Data Availability

The data presented in this study are available on request from the corresponding author.

## Abbreviations

ADP	Adenosine diphosphate
COFRAC	French Accreditation Committee
EMA	European Medicines Agency
FDA	Food and Drug Administration
LC–MS/MS	Liquid chromatography–mass tandem spectrometry
LLOQ	Lower limit of quantification
QC	Quality control
RE	Relative error
RSD	Relative standard deviation
RT	Retention time
